# Valedictory Address to the Graduating Class of the Atlanta Medical College of 1880

**Published:** 1880-03

**Authors:** J. A. Hurt

**Affiliations:** Hurtville, Ala.


### VALEDICTORY ADDRESS TO THE GRADUATING CLASS OF THE ATLANTA MEDICAL COLLEGE OF 1880.

By J. A. HURT. M.D., Hurtville, Ala.

*Fellow-Classmates, Ladies and Gentlemen:*

The occasion of this Assembly is one of solemity and pleasure peculiarly blended. It is solemn, because of the great responsibility which some present are about to assume: that of protecting human life against the thousand ills that flesh is heir to, and lengthening out the brittle thread of life. It affords pleasure by inspiring us with the hope, that those of us who assume this responsibility, may prove ourselves worthy of the trust that may be laid upon us; and this occasion will afford me great pleasure, if when it is over, I shall feel that I have done no injustice to the pride and anxiety of my colleagues, who have honored me with this appointment. Cared for, as we have been, by the noble enterprising men and women of Atlanta, we would do violence to our feelings, if we did not offer to them and to our corps of worthy instructors some expression of gratitude and praise for their sacrifice in our own behalf. To this, the "Gate City," and to the Atlanta Medical College, may it ever be an honor to claim us! To the Almuni and medical world, may there not be lacking some from this class who can stand as peers with the illustrious and honored members of the fraternity. May we spare no efforts to assure ourselves and convince the world that our labor is not in vain. And whether we die in vigorous manhood, while the battle is fierce, and while laudable ambition is unsatisfied, or when hoary age shall claim its own, may it be said of us, when we fall, that we fell at the post of duty.

Since the world began it has been the lot of man to suffer. This suffering is not propagated alone by vicissitudes of weather and climate, producing what we may term nature's legitimate offspring, but it is magnified and multiplied tenfold by our needless self-indulgencies, by our inordinate efforts to satiate an appetite pampered and fed by man's ingenuity. We eat and drink to excess; we toil and rest to excess; we love and hate to excess! Excess is marked upon all of our habits, and is a fruitful source of suffering. These excesses effect not only those who indulge, but remote results are handed down to succeeding generations, until in some instances they horrify a nation with suffering. But men may be temperate, prudent, and watchful, yet afflictions will come, and death will, sooner or later, summon them to the tomb! for it is appointed unto man once to die. The office of the physician is to endeavor to alleviate suffering and restore health to the diseased—to correct irregularities in development of both mind and body, and to assist nature in the performance of its proper functions. We seek to guard that boon, which is above price, the restoration of which has been sought in vain, by the untold wealth of kings. To embark in such a work is to assume a great responsibility. The science of medicine consists in the discovery and knowledge of disease, and the art of preparing and using the remedies for its removal. Notwithstanding a wise Creator has adjudged us deserving the punishment of disease and death, he has not left us without the means of alleviating pain and deferring the hour of dissolution. If there be balm in Gilead, if there be a physician there for moral diseases, it cannot be a thing unreasonable to allow men to lighten affliction, by searching in nature productions for a panacea. As an evidence of God's endorsement of our laudable work, we see everywhere on the globe something in nature that offers this relief, and a more peculiar fact is that the antidote and the disease generally subsist upon the same soil. From minerals which exist in rich abundance, all over the globe, and from numberless products of inanimate nature, we extract and combine elements whose properties are to counteract disease and restore health. True it is that in the roll of centuries diseases have multiplied, pain is no less acute, prudence and self denial are less practiced, and the appetites and passions are held in less submission to nature's laws; and if science did not come, with healing in its wings, the human race would soon become as lepers, "fit only to be cast without the gates of the city." How much then does the human race owe to such men as Dr. Crawford W. Long, the distinguished Georgian, who has done as much to alleviate the sufferings of frail humanity as any man living or dead. Suffering men and women will bless the name of the discoverer of anæsthesia to the remotest generations. Need we wonder then at the estimate placed upon the acquirement of correct medical knowledge and the skillful application of it? The very nature of its application demands a present and ready knowledge of its effects. The lawyer, after having a case presented, may leisurely converse with his client, and retire to his studio to study his case: the theologian sits in his sanctum, and days in advance arranges his thoughts for an occasion, but the physician is often called upon to confront diseases inevitably hazardous to life, which demand immediate rest in order to stay the hand of the dertroyer. And he who stands and waits for stern necessity to move him, will, in the hour of greatest trials, find that he is utterly devoid of the elements of success, and of the power to stay the storm which is soon to separate mortality from immortality; and finally in the sad moment, when the silent tomb lays claim to its victim, he writhes under the consciousness of failure in duty. While mourning friends weep over the loss sustained, he recounts the hours of idleness, which, had they been profitably spent, might have enabled him, in the providence of God, to alter the result. The Doctor is not infallible, but if he be ready and properly panopolied for the fight, he may often bridge the chasm and foster the strength of his patient. "Industrious wisdom often does prevent what lazy folly thinks inevitable." Nature, though ever on the alert, and striving to fortify against the attacks of disease, is often overpowered, and without the assistance of him who has acquainted himself with the healing art, it must succumb.

Fellow-comrades, we have chosen a profession that is elevating, and though appalling in its responsibilities, it is deeply fascinating and worthy the devotion of a lifetime. If we will pursue it hereafter in earnestness and truth, it will probably conduct us to an honorable competence, and will, most assuredly, prove a source of great mental and moral discipline. It is conversant with objects which tend to elevate the thoughts, temper the feelings, touch the heart, affording ample scope and exercise for the intellect. We have before us a broad field, to cultivate in behalf of humanity; and since by professional duties, we shall be thrown in contact with different classes of mankind, the rich and the poor, the cultivated and the untutored, and necessarily learn more or less of the character and habits of our patrons, we shall be called upon to exercise charity and benevolence, to inform those who are uninformed, especially as regards hygiene, and to offer sympathy and condolence to those who are suffering and bereaved. Our counsel will often be sought in matters which involve both legal and moral questions. Upon our testimony may depend the happiness or misery of a family; upon .our medical opinions may rest the fate of some one accused of crime. Upon our knowledge and reasoning may rest the turning point of averting some dreadful scourge which might devastate entire cities, and make them one grand mass of decomposition. By our conduct and conversation, while in the discharge of professional duty since we gain the confidence of the family and become acquainted with many family matters, the feelings and thoughts of our patrons may be directed for weal or for woe. While we are not held in supreme reverence and are not looked upon as infallible, it is expected of us that we hold in utter disdain any act tending to disgrace humanity, and to attain to and hold an exalted standpoint: that we lay up in store that knowledge which will serve us in the chamber of the sick, and capacitate and nerve us to combat diseases successfully. Fortitude in the trying times of our lives can only be maintained under the consciousness of having previously equipped ourselves to meet the assailant. When anxiety and terror seize the friends, and excitement bewilders the nurses, while they look upon the patient in impending death, we are to be cool and deliberate, and guide with a steady hand the instrument of relief, and direct without hesitation the administration of the remedy. Then let us consider well these grave responsibilities, and beware how we idly neglect or selfishly abuse a stewardship so precious and yet so weighty. Let us not dare, without due consideration, to embark in a calling such as this, so capable of good, if rightly used, so full of peril to ourselves and to mankind, if administered ignorantly and unfaithfully.

"Let us not be like the empiric ant, who collects from every side indiscriminately, for present wants, nor speculative like the spider, who spins his web of sophistry from the recesses of his inner being, but imitate the praiseworthy bee, who gathering crude honey from various flowers, stores it up within and by his own operations matures and perfects it for future use." Ambition, worldly honor, love of gain, all may have their parts in impelling us to success, and in their places, being well tempered, they may be valuable elements, but let no charlatans, who "float simply from corruption and only rise as they rot," pervade our ranks. The man who gains the confidence of the affiliated by false pretenses and lives by preying upon the miseries of mankind, has stolen the palladium from the temple to entail countless ills upon the human race. It is here you have laid the foundations upon which to build your future structures, each to stand forth in strength and beauty representing the labors bestowed upon it. Let your lives be marked with manliness, industry, prudence and perseverance. Straight as the tempered steel directs its course north, press onward. Let thought be in thine eye and from thy brow the dew of labor start.

And now to our honored professors, let me on leaving you, in behalf of my classmates, return you our heartfelt thanks, for your zeal in our behalf. You have taught us faithfully and in love; we are pained at parting from you, but we must to the work you have bid us, stopping only to bid you farewell. And again to my classmates, allow me to say, that our intercourse has been most pleasant and profitable. We shall not forget our stay in Atlanta; we shall not forget our friends here; we shall not forget our Alma-Mater; we shall, no doubt, often recur to the days spent here, as the halcyon days of life. Time and distance will soon separate us. This day marks the dividing line between youthful dreams and manhood's realities. We now enter the arena of active life, and shall we forget each other? I trust not. And my parting wish for you and everyone of you is, that you may be successful as physicians, and become eminent in the profession. And now ladies and gentlemen, although the pleasure of this occasion is marred by the sad reality that we are so soon to separate from such dear friends, I hail with pleasure this opportunity of expressing to you our thanks, for the kind attention shown us during our sojourn in this city. And I could wish for you no greater happiness, than that every day of your lives, may be as happy as you have made for us each day of our stay in your midst.

May we in future prove ourselves worthy of these kindnesses, but we must part and hence—farewell!

